# A Peculiar Case of Idiosyncrasy

**Published:** 1892-08

**Authors:** B. C. Powell

**Affiliations:** Villa Rica, Ga.


### *CORRESPONDENCE.*

---

VILLA RICA, GA., July 2, 1892.

*Editor Atlanta Medical and Surgical Journal :*

SIR—I wish to report to your readers the following case of a peculiar idiosyncrasy which has recently come to my notice:

Miss R. D., age twenty-five, teacher in Villa Rica High School, general health good, monthlies regular. She came here from Senoia, Ga., where she was born and reared. Has no trouble except when she violates one law that dame nature has given her, and that is she must not eat wheat flour nor anything that contains the smallest quantity of it. Not because it interferes solely with digestion but that it acts as a irritant poison to the mucous membrane of mouth, throat and stomach. The pain is so great, retching and vomiting so severe as to almost cause convulsions. She was almost killed time and again when small, before her parents found that she could not eat wheat flour. People with whom she would visit, thinking it only a silly idea of hers, would attempt to fool her by mixing small quantities of wheat flour with other articles of food and get her to eat of it, but she would invariably suffer for it. When she first came here she began boarding with Dr. G. W. Strickland (dentist), and at one time his wife, though knowing the young lady's peculiarity, had some muffins of corn meal made up in a tray that was used for flour, but put no flour with it except what little was sticking to the tray. At supper the young lady ate of the muffins and could not tell by taste that they had any flour whatever about them, but pretty soon she was seized with pain, vomiting and the usual symptoms of poisoning, and when the tray was thought of the trouble was accounted for, though a physician had to be called before relief could be obtained. No other article of food acts in any way harmfully, therefore it must not be the starch in the flour, for we know there is plenty of starch in corn, potatoes, etc., all of which she can eat with impunity. We know this patient is possessed of a very peculiar idiosyncrasy, but the cause of it is still a mystery and difficult to account for.

Will some member of the profession advance a theory in regard to the cause of such an idiosyncrasy?

Dr. G. W. Strickland and S. C. Dobbs & Co., druggists, will corroborate the above facts.

B. C. POWELL, M. D.

